# A first-in-class precision antibody conjugate targeting EGFR, mTOR, and PI3K to treat head and neck cancers

**DOI:** 10.21203/rs.3.rs-9655840/v1

**Published:** 2026-05-14

**Authors:** Xiaoyi Li, Meghri Katerji, Lily M. Klapper, Siddharth Matikonda, Nick Brill-Edwards, Aamna Siddiqui, Chathuranga Rathnamalala, Mo Yang, Jungwuk Lee, Ryan Bensen, Lai Thang, Simone Difilippantonio, Julien Dugal-Tessier, Shengzong Liang, John Brognard, Martin Schnermann

**Affiliations:** 1 Chemical and Biological Laboratory, National Cancer Institute, National Institutes of Health, Frederick, MD 21702.; 2 Department of Surgery, Pharmacology, and Biochemistry and Molecular Biology, State University of New York, Upstate Medical University, Syracuse, NY 13210.; 3 Cancer Therapeutics Program, State University of New York, Upstate Medical University, Syracuse, NY 13210.; 4 Laboratory of Cell and Developmental Signaling, National Cancer Institute, National Institutes of Health, Frederick, MD 21702.; 5 Animal Technical Support, Laboratory Animal Sciences Program, Leidos Biomedical Research, Frederick National Laboratory for Cancer Research, Frederick, MD 21702.; 6 NJ Bio Inc., Princeton, NJ 08540.

## Abstract

Head and neck squamous cell carcinoma (HNSCC) exhibits limited response to EGFR blockade with cetuximab, largely due to constant activation of the PI3K/AKT/mTOR pathways. Despite the development of numerous PI3K/mTOR inhibitors, their clinical application remains constrained by dose-limiting on-target toxicities. Here we report PAC-XL, a precision antibody conjugate linking the PI3K/mTOR inhibitor BGT226 to cetuximab through a β-glucuronidase-cleavable benzyl-ammonium carbamate (BAC) linker. Unlike conventional linkers, the BAC chemistry enabled a homogeneous, high drug-to-antibody ratio (DAR=8) conjugate that preserved EGFR binding, antigen-mediated uptake, lysosomal trafficking, plasma stability, and enzyme-dependent payload release. PAC-XL induced EGFR- and PI3K/mTOR-dependent cytotoxicity, suppressed PI3K/mTOR signaling, and triggered apoptosis in PIK3CA-altered HNSCC models. In xenografts, PAC-XL outperformed cetuximab, BGT226 and alpelisib, including complete regressions, while reducing hyperglycemia and weight loss caused by systemic PI3K/mTOR inhibition. These findings establish targeted delivery of PI3K/mTOR inhibitors as a strategy to enhance efficacy while improving tolerability in HNSCC.

## Introduction

Head and neck squamous cell carcinoma (HNSCC) is a prevalent epithelial cancer of the oral cavity, pharynx, and larynx.^1^ Epidermal growth factor receptor (EGFR) signaling is a major oncogenic driver in this disease and EGFR is overexpressed in roughly 80% of tumors.^2^ Cetuximab, an EGFR-targeting monoclonal antibody, is approved for recurrent or metastatic HNSCC, and immune checkpoint blockade with pembrolizumab or nivolumab has become standard of care for many patients with platinum-refractory disease.^3^ However, durable benefit remains limited to a subset of patients, highlighting the need for therapies that are both effective and tolerable.^4^

Beyond EGFR, other pathways support HNSCC growth and therapeutic escape.^5^ Alterations such as PIK3CA gain-of-function (GOF) mutations or amplifications and PTEN loss drive constitutive PI3K/AKT/mTOR signaling in the majority of HNSCC cases.^6^ Upregulated PI3K/mTOR activity can also promote both intrinsic and acquired resistance to cetuximab.^7^ PI3K inhibitors (e.g., alpelisib or copanlisib) when combined with cetuximab have demonstrated efficacy in preclinical HNSCC models, however these combinations have not delivered consistent therapeutic benefit.^8^ A central barrier is dose-limiting, on-target toxicity from PI3K signaling pathway inhibition in normal tissues, including hyperglycemia and gastrointestinal toxicity. Consequently, there is a need for approaches that engage canonical HNSCC dependencies while improving the therapeutic window.

Antibody-drug conjugates (ADCs) seek to increase tumor specificity by coupling antibodies to small molecule therapeutics.^9^ Clinically deployed payloads largely fall into a small set of mechanisms – microtubule disruption, DNA damage, and, more recently, topoisomerase inhibition.^10,11^ While highly effective, ADC-associated toxicities have remained a significant barrier to broader clinical adoption.^12^ Current efforts to improve therapeutic outcomes include rational combinations with kinase inhibitors or immune checkpoint blockade, and expansion of payload classes to include small-molecule inhibitors and targeted protein degraders that are now entering clinical development.^13^ A major limitation to broadening payload classes is linker chemistry. Many widely used linkers are poorly suited for hydrophobic sp-2-rich payloads – a property of many kinase inhibitors – restricting achievable drug-to-antibody ratios (DARs) and impacting payload release.^14,15^ Next-generation linkers that enable efficient conjugation of emerging payload classes, while maintaining circulation stability and controlled release are essential to expand the scope of antibody-directed therapies.

**Fig. 1 ∣:**
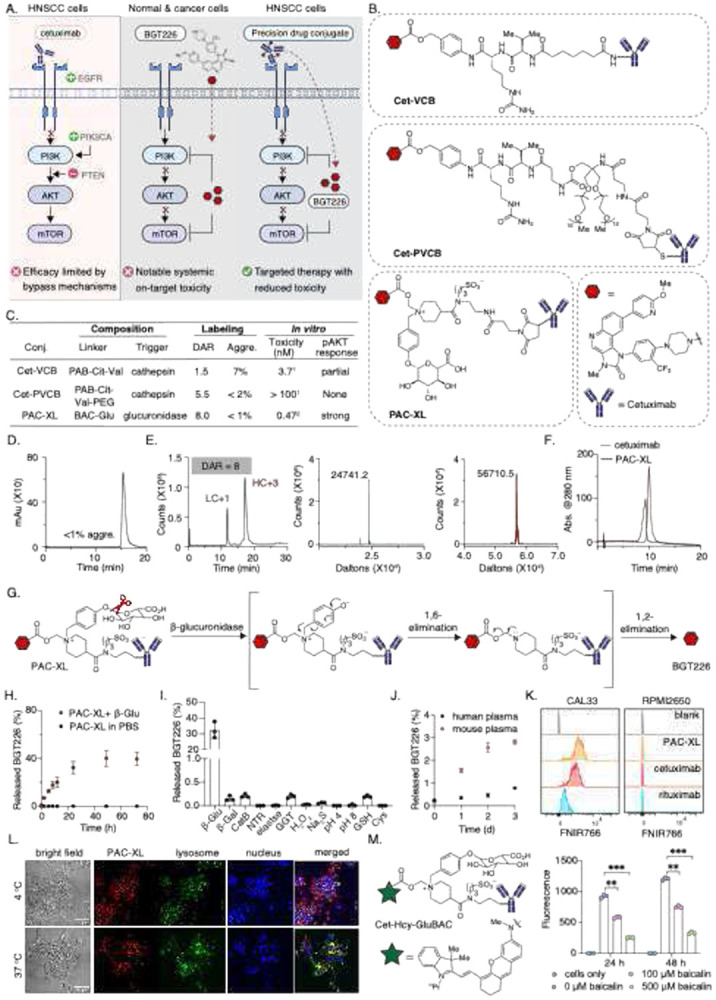
Generation and *in vitro* characterization of PAC-XL. (A) Schematic illustration of the mechanisms of action of cetuximab, BGT226, and the precision antibody conjugate PAC-XL. (B) Evolution of linker design leading to identification of the optimal linker architecture for PAC construction. (C) Overview of the three generations of PAC constructs, summarizing their molecular composition, labeling properties, and *in vitro* performance. (D) FPLC analysis of PAC-XL. (E). HPLC–QTOF–MS analysis of reduced PAC-XL. LC: light chain of the conjugate; HC: heavy chain of the conjugate (F) HIC analysis of PAC-XL. (G) Proposed 2-step cleavage chemistry of the benzyl-ammonium carbamate linker. (H) *In vitro* BGT226 release from 5 μM (payload concentration) PAC-XL with 5 KU/mL β-glucuronidase over 72 h. (I) *In vitro* BGT226 release from 5 μM (payload concentration) PAC-XL in the presence of different physiological triggers after 24 h. (J) Stability assessment of PAC-XL in 50 % human plasma and complete mouse plasma at 37 °C over 72 h. (K) Representative flow cytometry analysis (mode normalized) for the binding of cetuximab, PAC-XL and rituximab in CAL33 (high EGFR expression) and RPMI2650 (low EGFR expression) cells. Conjugates were labelled with FNIR-766 and incubated with cells for 24 h. N=3. (L) Confocal imaging analysis of the binding and internalization of PAC-XL. PAC-XL was labelled with FNIR-766 and incubated with CAL33 cells for 4 h at 4 °C or 18 h at 37 °C. scale bar = 50 μm. (M) Assessment of the release of BGT226 in CAL33 cells by cellular β-glucuronidase using a fluorogenetic PAC-XL analogue. For H-J, M, data are shown as mean ± SD (n = 3). Statistical analyses were performed by unpaired t-test with Prism (**p < 0.01, ***p < 0.001).^1^CAL33 cells were treated with Cet-VCB or Cet-PVCB for 3 days, and cytotoxicity was assessed using MTS assays. ^2^Cells were treated with PAC-XL for 5 days, and cytotoxicity was evaluated using alamarBlue assays. N=3.

In this work, we introduce precision antibody conjugates (PACs) as a new therapeutic modality which leverages antibody-based delivery of signaling inhibitors aligned with tumor-specific dependencies. Our goal is (1) enhance the efficacy of targeted therapies through coordinated inhibition of multiple oncogenic pathways and (2) mitigate the on-target toxicities associated with systemic exposure to small-molecule inhibitors. HNSCC provides an ideal setting to evaluate this approach as the clinical activity of cetuximab is limited by the hyperactivation of the PI3K/mTOR pathway. To co-target EGFR and PI3K-mTOR, we envisioned combining cetuximab and BGT226, a dual PI3K/mTOR inhibitor that displays potent *in vitro* activity across solid tumors and hematologic cancer models,^16-18^ however was discontinued in phase I/II clinical trials due to systemic toxicity^16,19^ (Fig. 1a). Through iterative optimization of linker chemistry (Fig. 1b), we identified a benzyl-ammonium carbamate (BAC) linker recently described by our group, ^20^ which, when combined with a β-glucuronide trigger, enabled the generation of PAC-XL with high drug-to-antibody ratio (DAR), minimal protein aggregation and efficient payload release. Consistent with its design, PAC-XL exhibited superior antitumor activity compared with cetuximab in both HNSCC cell-based assays and xenograft models, and was well tolerated *in vivo*, without inducing hyperglycemia or body weight loss at high doses.

## Results

**Extended Data Fig. 1 ∣:**
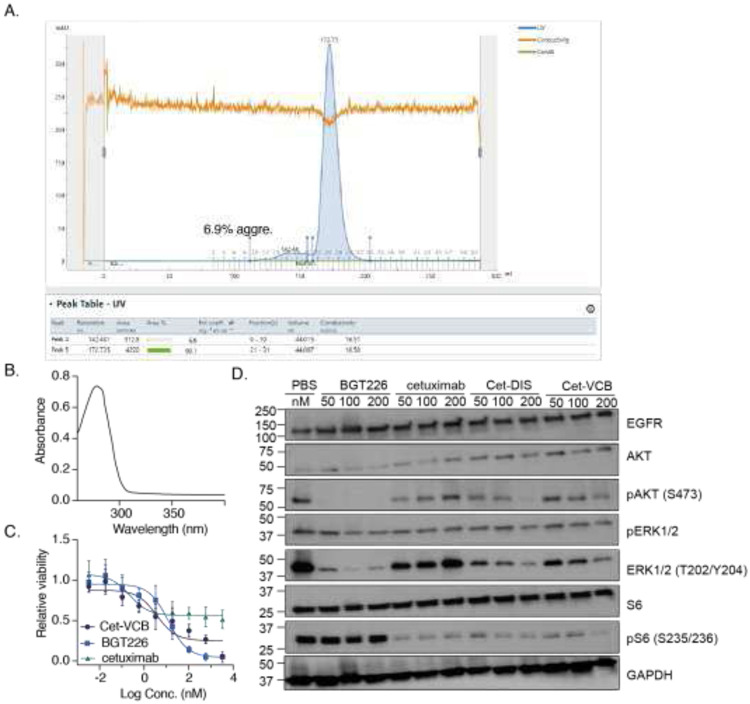
Characterization of Cet-VCB. (A) FPLC spectrum of Cet-VCB; (B) absorption spectrum of Cet-VCB in PBS (10 mM, pH 7.2); DAR was calculated according to the absorbance at 280 nm (antibody) and 340 nm (payload); cytotoxicity (C) and western blot analysis (D) of Cet-VCB, BGT226 and cetuximab in CAL33 cells. Cet-DIS is an analog of Cet-VCB in which the Val–Cit linker is replaced with a disulfide linker.

To identify a linker compatible with the hydrophobic dual PI3K/mTOR inhibitor BGT226, we used an iterative approach that evaluated both conventional standard and emerging chemistries. We first evaluated a conventional self-immolative *p*-aminobenzyl (PAB) spacer combined with a cathepsin-cleavable Val–Cit dipeptide. Coupling this Val–Cit–PAB module to the piperazine of BGT226 and conjugating to cetuximab via amide formation yielded Cet-VCB (Fig. 1b; synthesis described in supporting information). Cet-VCB showed appreciable aggregation (7%) and a low DAR (1.5) (Fig 1c). These suboptimal physicochemical properties corresponded with modest cytotoxicity in HNSCC cell line CAL33 and only partial inhibition of pAKT signaling (Extended Data Fig. 1), and early *in vivo* studies likewise produced limited antitumor responses.

**Extended Data Fig. 2 ∣:**
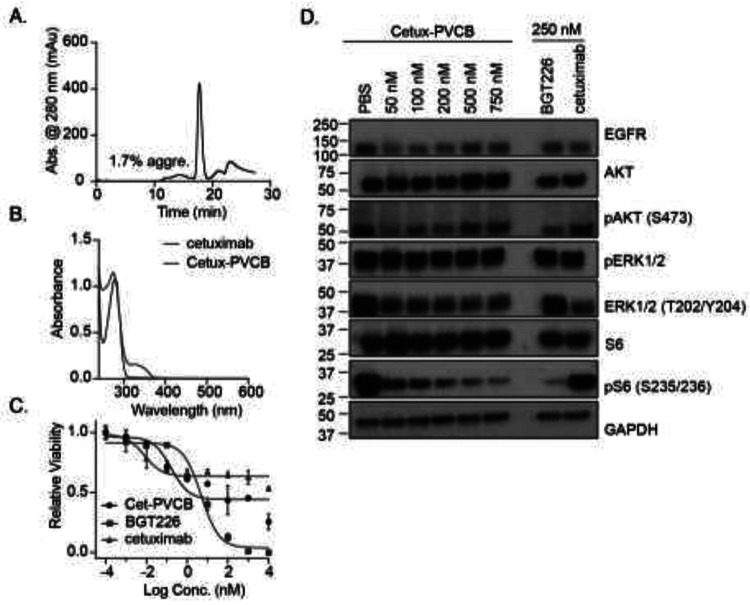
Characterization of Cet-PVCB. (A) FPLC spectrum of Cet-PVCB; (B) absorption spectrum of Cet-PVCB and cetuximab in PBS (10 mM, pH 7.2); DAR was calculated according to the absorbance at 280 nm (antibody) and 340 nm (payload); cytotoxicity (C) and western blot analysis (D) of Cet-PVCB, BGT226 and cetuximab in CAL33 cells.

To reduce hydrophobicity, we incorporated a branched 24-unit PEG domain into the Val–Cit–PAB linker and used maleimide-cysteine chemistry for conjugation (Cet-PVCB; Fig. 1b). This construct achieved markedly lower aggregation (<2%) and a higher DAR (5.5) (Fig. 1c). However, in CAL33 cells, its activity was indistinguishable from unconjugated cetuximab, and it did not produce concentration-dependent suppression of pAKT or downstream signaling (Extended Data Fig. 2). While PEGylated linkers can improve conjugate properties in other contexts, these data suggest that the PEG domain can hinder productive enzymatic activation or self-immolation for BGT226 in this setting.^21,22^

**Extended Data Fig. 3 ∣:**
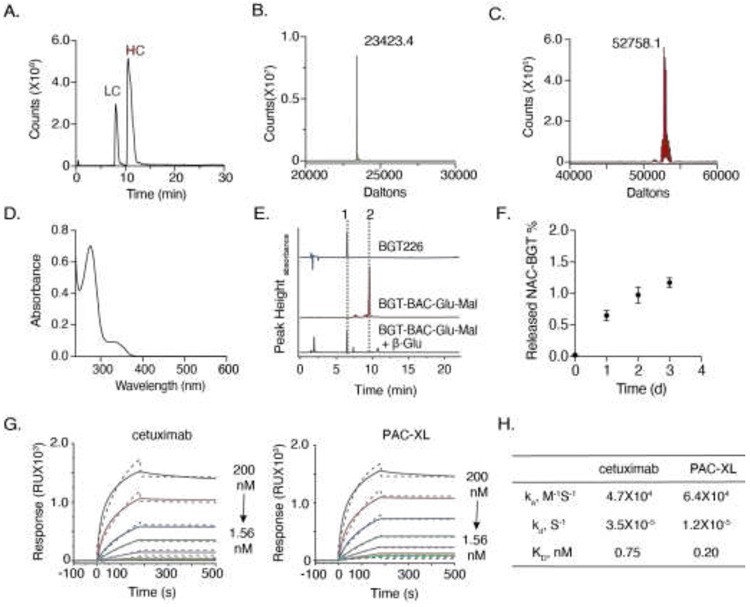
Additional characterization of PAC-XL. (A) Total ion chromatogram of reduced cetuximab; deconvoluted mass spectra of (B) light chain (LC) and (C) heavy chain (HC) of reduced cetuximab. The averaged DAR for PAC-XL was determined to be 8 across multiple batches. (D) Absorption spectrum of PAC-XL in PBS (10 mM, pH 7.2). DAR was calculated according to the absorbance at 280 nm (antibody) and 340 nm (payload). The averaged DAR was 8 across multiple batches, consistent with the HPLC–QTOF–MS result. (E) HPLC traces of 50 μM BGT226 in PBS (top); 50 μM BGT-BAC-Glu-Mal in PBS (middle); and the reaction products of 50 μM BGT-BAC-Glu-Mal with 500 U/mL β-glucuronidase in PBS (bottom) for 3 h at 37 °C. Assignments of the peaks: (1) 6.5 min, BGT226; (2) 9.6 min, BGT-BAC-Glu-Mal. The eluted peaks were monitored at 280 nm. (F) HPLC–QTOF–MS analysis of the retro-Michael deconjugation for PAC-XL in PBS at 37 °C over 72 h. Data are shown as mean ± SD (n = 3). (G) SPR sensorgram of cetuximab and PAC-XL to human EGFR immobilized surface; dashed lines for raw binding curves; solid lines for fitted curves; (H) corresponding k_a_, k_d_, and K_D_.

Given the limitations of both Val–Cit designs, we next evaluated our benzyl-ammonium carbamate (BAC) linker platform, which increases hydrophilicity and enables payload release through a unique two-step cleavage cascade.^20^ After screening multiple trigger chemistries, we tested β-glucuronidase-cleavable variants, which augment the hydrophilicity of the linker and build upon their prior use in ADC development.^23^ Initial characterization of the labeling chemistry revealed that this combination provided an ADC, PAC-XL, that consisted almost entirely of a monomeric species, as confirmed by fast protein liquid chromatography (FPLC) (>99% purity; Fig. 1b, 1c, 1d, synthesis described in the supporting information). High-performance liquid chromatography-quadrupole time-of-flight mass spectrometry (HPLC–QTOF–MS) analysis of reduced cetuximab and PAC-XL indicated an average DAR of 8.0 across multiple batches (Fig. 1e, Extended Data Figs. 3a-3d) and mass spectra data corresponding to one payload on each light chain and three payloads on each heavy chain. Hydrophobic interaction chromatography (HIC) revealed a single peak with only a modest increase in hydrophobicity relative to cetuximab (Fig. 1f). Together, these data indicate efficient conjugation of BGT226 to cetuximab through the glucuronidase-cleavable BAC linker, to produce homogeneous, high-DAR construct.

**Extended Data Fig. 4 ∣:**
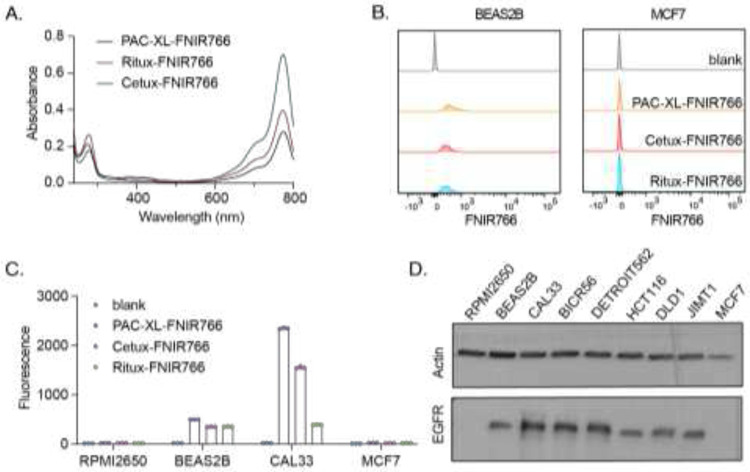
Binding analysis of PAC-XL. (A) Absorption spectra of PAC-XL-FNIR766, Cetux-FNIR766 and Ritux-FNIR766 in PBS (10 mM, pH 7.2). DAR was calculated according to the absorbance at 280 nm (antibody), 340 nm (BGT226) and 766 nm (FNIR766). The degree of FNIR766 labeling was calculated as 2.7, 2.6 and 1.7 for PAC-XL, cetuximab and rituximab, respectively. (B) Representative FlowJo histograms showing fluorescence intensity in the APC-Cy7 channel for BEAS2B and MCF7 cells. PAC-XL, cetuximab and rituximab were labelled with FNIR766 and incubated with cells for 24 h. *λ*_ex_ = 637 nm, *λ*_em_ = 750 – 810 nm. (C) Quantitative analysis of the binding and uptake of PAC-XL, cetuximab and rituximab in different lines. N=3. (D) Western blot analysis of the EGFR expression level in different cells.

**Extended Data Fig. 5 ∣:**
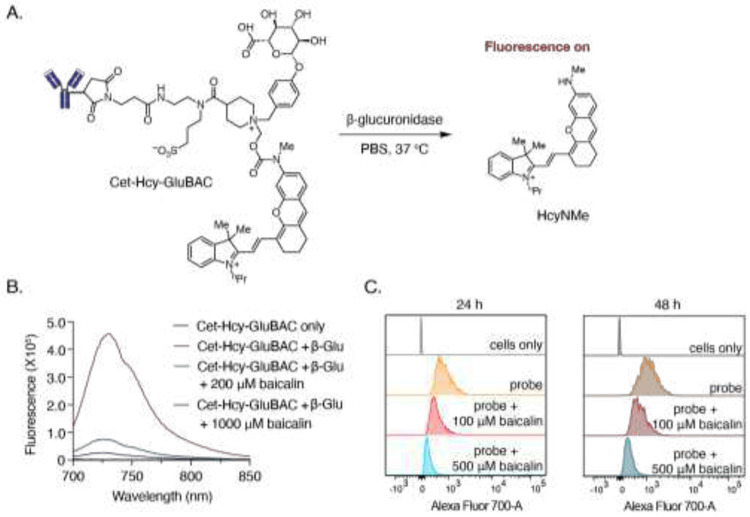
Release of fluorogenic PAC-XL analogue under β-glucuronidase treatment. (A) Fluorescence turn-on mechanism of Cet-Hcy-GluBAC. (B) Fluorescence spectra of 100 μg/mL Cet-Hcy-GluBAC before and after reaction with β-glucuronidase (2.5 KU/mL) in the presence or absence of baicalin for 24 h in PBS (10 mM, pH 7.2). *λ*_ex_ = 690 nm, *λ*_em_ = 700 – 850 nm. (B) Flow cytometry analysis of the release of HcyNMe fluorescent payload in CAL33 cells treated with Cet-Hcy-GluBAC (50 μg/mL) in the presence or absence of baicalin (100 μM or 500 μM) at 24 h and 48 h. Representative mode-normalized FlowJo histograms showing fluorescence intensity in the Alexa Fluor 700 channel. *λ*_ex_ = 637 nm, *λ*_em_ = 685 – 735 nm.

The glucuronide-modified BAC linker is designed to release payload through β-glucuronidase cleavage followed by sequential 1,6- and 1,2-elimination to regenerate free BGT226 (Fig. 1g). Consistent with this mechanism, HPLC confirmed BGT226 release from a small-molecule analogue upon β-glucuronidase treatment (Extended Data Fig. 3e), and HPLC–QTOF–MS showed ~30% payload release from PAC-XL after 24 h in 5 KU/mL β-glucuronidase (Fig. 1h). In contrast, incubation with other common ADC triggers and physiological species did not yield appreciable release (Fig. 1i), supporting β-glucuronidase specificity. PAC-XL was found to be stable in plasma, with <1% total payload release in 50% human plasma and <3% release in mouse plasma after 72 h (Fig. 1j). Retro-Michael deconjugation of the thiosuccinimide linkage was evaluated by trapping released maleimide with N-acetylcysteine; HPLC–QTOF–MS analysis indicated deconjugation remained below 2% after 72 h in PBS (Extended Data Fig. 3f).^24^ PAC-XL retained EGFR binding with SPR measurements against recombinant human EGFR showing comparable affinities to cetuximab alone (K_D_ = 0.20 nM for PAC-XL vs. 0.75 nM for cetuximab; Extended Data Figs. 3g, 3h). Flow cytometry using FNIR-766-labeledPAC-XL and cetuximab revealed fluorescence intensities that correlated with EGFR expression across cell lines, while the non-targeting antibody rituximab produced minimal signal, confirming EGFR-specific binding (Fig. 1k, Extended Data Fig. 4).^25^ Spinning-disk confocal microscopy demonstrated efficient internalization and lysosomal trafficking of PAC-XL (Pearson correlation coefficient = 0.87; Fig. 1l). To evaluate intracellular linker activation, a fluorogenic PAC analogue (Cet-Hcy-GluBAC) was prepared by replacing BGT226 with a hemi-cyanine payload whose fluorescence is restored upon β-glucuronidase cleavage (Fig. 1m, Extended Data Fig. 5).^20^ CAL33 cells incubated with Cet-Hcy-GluBAC exhibited strong fluorescence that was markedly reduced by baicalin, a β-glucuronidase inhibitor, supporting enzyme-dependent cellular activation. Collectively, these data suggest PAC-XL combines plasma stability with trigger-dependent release and antigen-mediated uptake.

**Extended Data Fig. 6 ∣:**
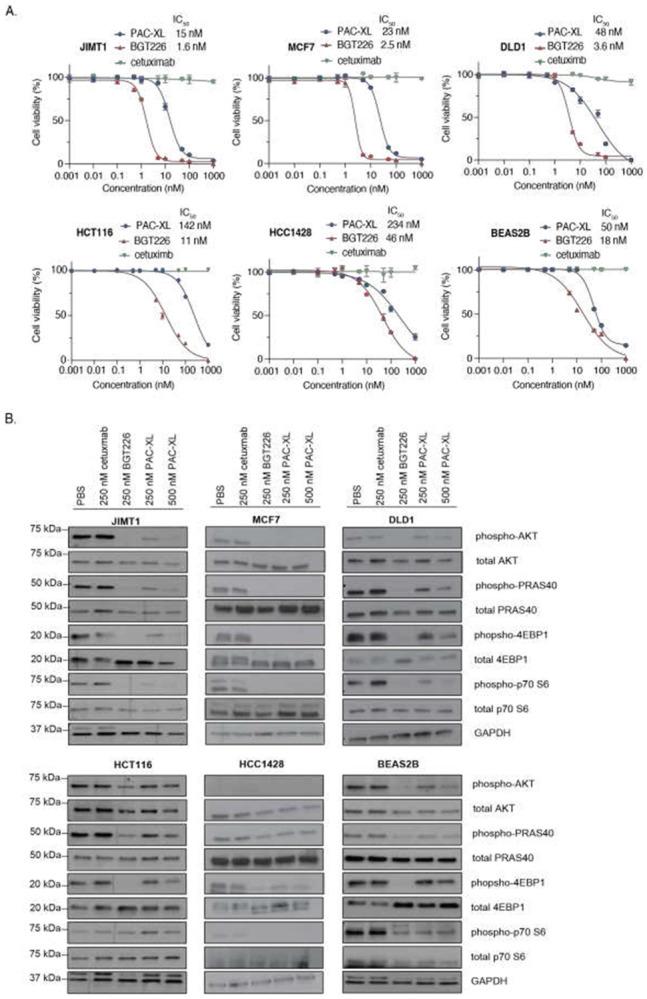
Cellular responses of PAC-XL. (A) Cytotoxicity curves of PAC-XL, cetuximab and BGT226 in different cell lines. Cells were incubated with different concentrations of PAC-XL, cetuximab and BGT226 for 5 days. AlamarBlue assay was used to determine the cell viability. Data points are shown as mean ± SD (n = 3). Data analysis was performed in GraphPad Prism10 using the non-linear regression (four-parameter) algorithm. (B) Representative Western blot analysis of PAC-XL, cetuximab, and BGT226 in different cell lines after 24–h treatment.

**Fig. 2 ∣:**
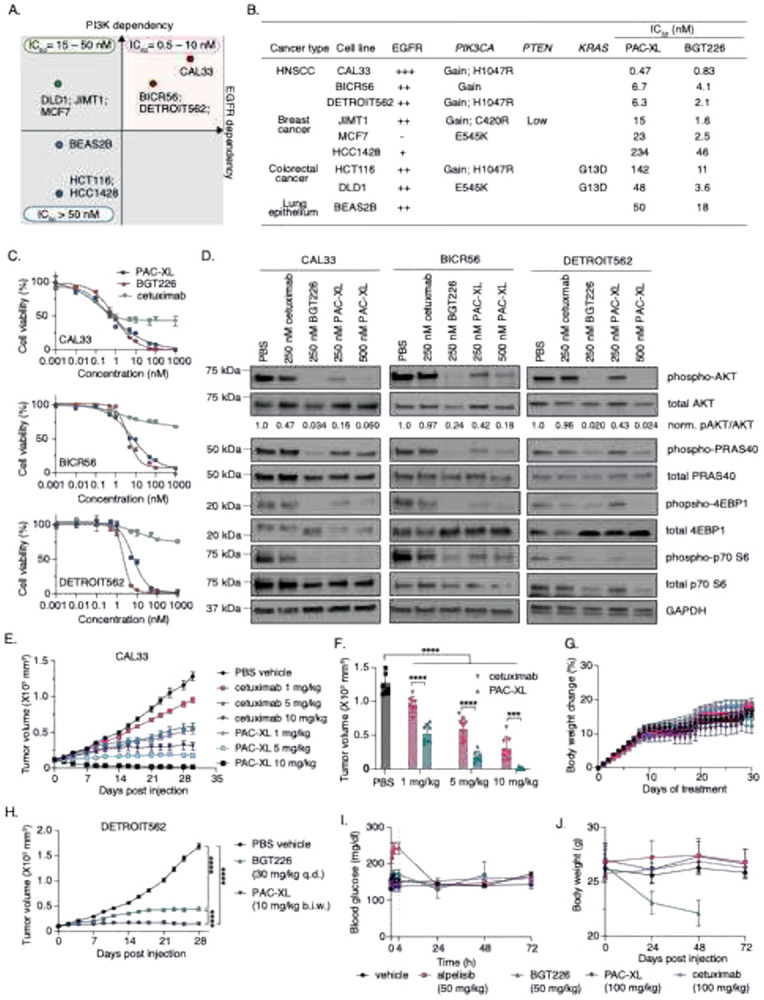
Evaluation of PAC-XL Cellular and *In Vivo* Activity. (A) General classification of tested cell lines. (B) Summary of cytotoxicity data of PAC-XL and BGT226 across a panel of cell lines with relevant genetic alterations. (C) Cytotoxicity and (D) western blot results of PAC-XL, cetuximab and BGT226 in three HNSCC lines. Data are shown as mean ± SD (n = 3). (E) Tumor growth curves, (F) tumor volumes measured on the last day and (G) corresponding body weight changes in CAL33 xenograft-bearing mice treated with PBS, cetuximab, or PAC-XL at 1, 5 and 10 mg/kg, administered i.p. twice weekly. Data points are displayed as the mean ± SEM (n = 8). (H) Tumor growth curves in DETROIT562 xenograft-bearing mice. BGT226 was administered i.p. once daily at 30 mg/kg; PBS and PAC-XL were administered i.p. twice weekly at 10 mg/kg. Data points are displayed as the mean ± SEM (n = 10). Statistical analysis of final tumor volumes was performed across all groups using one-way ANOVA in Prism. ***p < 0.001, ****p < 0.0001. (I) Blood glucose levels and (J) body weight changes in CD-1 mice following i.p. treatment with alpelisib (50 mg/kg), BGT226 (50 mg/kg), PAC-XL (100 mg/kg), or cetuximab (100 mg/kg). Data points are displayed as the mean ± SEM (n = 3).

PAC-XL was profiled across a panel of human cancer cell lines. Based on responses to cetuximab and BGT226, these cell lines can be stratified into three groups: (1) EGFR-dependent and PI3K-dependent; (2) EGFR-independent but PI3K-dependent; and (3) EGFR-independent and PI3K-independent (Figs. 2a, 2b). HNSCC lines with dual EGFR and PI3K dependence (CAL33, BICR56, and Detroit562) were most sensitive to PAC-XL. These models harbor PIK3CA GOF mutations or amplifications associated with hyperactivation of the PI3K–mTOR pathway which promotes intrinsic resistance to cetuximab. PAC-XL displayed high nanomolar potency (IC_50_ = 0.47–6.7 nM), comparable to unconjugated BGT226 (Fig. 2c). Consistently, it robustly suppressed PI3K–mTOR signaling in a concentration-dependent manner (Fig. 2d), as evidenced by the reduced phosphorylation of AKT and downstream mTOR targets (p70S6K and 4E-BP1), indicating efficient intracellular delivery and pathway engagement . In other models, the activity of PAC-XL decreased as a function of EGFR and PI3K dependence. As shown in Extended Data Fig. 6, in EGFR-independent but PI3K-dependent cells (JIMT1, MCF7 and DLD1), PAC-XL exhibited reduced potency (IC_50_ = 15–50 nM) and attenuated pathway suppression relative to free BGT226. The moderate cytotoxicity of PAC-XL in EGFR-low MCF7 cells (IC_50_ = 23 nM) likely reflects extracellular linker cleavage. In contrast, EGFR- and PI3K-independent lines (BEAS2B, HCT116, and HCC1428) were insensitive to both cetuximab and BGT226 and accordingly showed minimal response to PAC-XL (IC_50_ = 50–234 nM) with limited suppression of downstream signals. These differences are consistent with distinct drivers of pathway independence (e.g., KRAS activation in colorectal lines, hormone dependence in HCC1428 lines and intact growth control in the immortalized bronchial epithelial cell line, BEAS2B). Overall, PAC-XL cytotoxicity is a function of both EGFR targeting and PI3K pathway dependence, suggesting PACs can be more selective than conventional ADCs with broadly cytotoxic payloads while addressing cetuximab resistance pathways.

**Extended Data Fig. 7 ∣:**
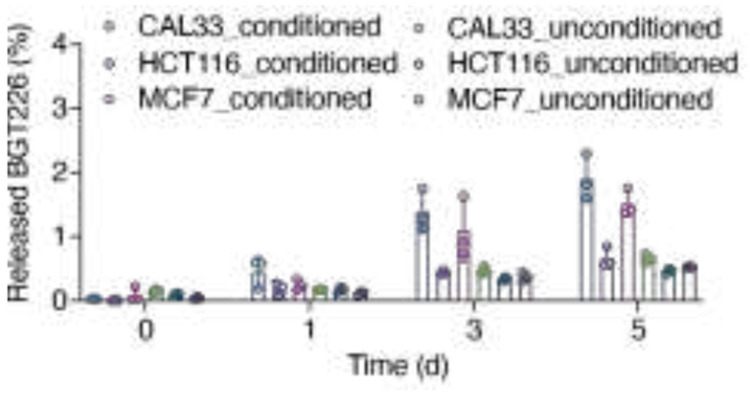
Investigation of the extracellular release of PAC-XL. HPLC–QTOF–MS analysis of extracellular release of BGT226 from PAC-XL in conditioned cell culture media collected from CAL33, HCT116 and MCF7 cells. PAC-XL was incubated with conditioned or unconditioned media at 37°C for 1–5 days. Data are shown as mean ± SD (n = 3).

We then assessed the contribution of extracellular β-glucuronidase activity. β-Glucuronidase localizes to lysosomes but can also be secreted into the extracellular milieu, where it may mediate linker cleavage.^26,27^ To test this, conditioned media was collected from multiple cell lines and HPLC–QTOF–MS analysis was used to determine the levels of released BGT226. Consistent with the cytotoxicity data described above, higher levels of cleavage were observed in media from MCF7 and CAL33 cells compared to HCT116 cells (Extended Data Fig. 7). Extracellular release has been reported for multiple cleavable linker classes, including Val–Cit and β-glucuronide.^28,29^ This property may be beneficial by leading to localized drug release within the tumor microenvironment, enabling bystander killing of neighboring tumor cells regardless of antigen expression.

**Extended Data Fig. 8 ∣:**
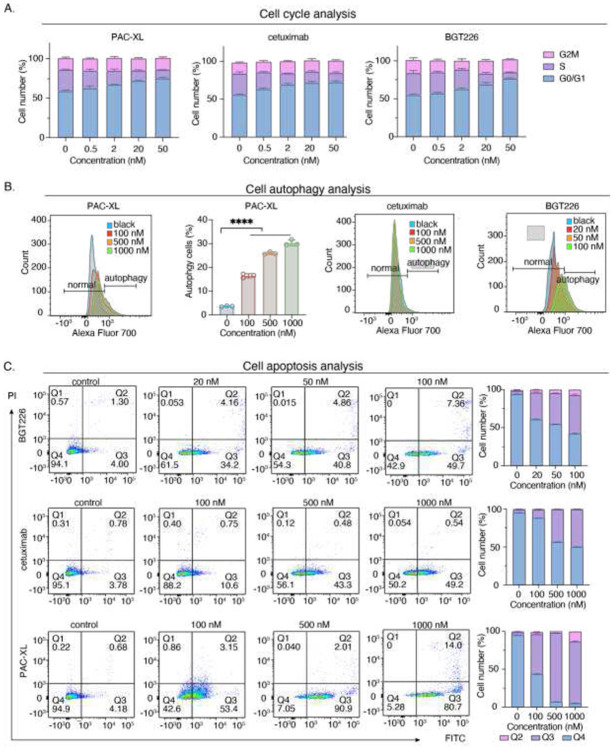
Cell killing mechanisms of PAC-XL. (A) Cell cycle analysis of CAL33 cells treated with PAC-XL, cetuximab and BGT226 (0–50 nM) for 48 h. Data points are displayed as the mean ± SD (n=3). (B) Flow cytometry analysis of the autophagy in CAL33 cells after 72 h: representative FlowJo histograms showing fluorescence intensity in the Alexa Fluor 700 channel of cells treated with PAC-XL, cetuximab and BGT226, and quantitative analysis of geometric mean fluorescence intensity from PAC-XL treatment. Data are shown as mean ± SD (n = 3). Statistical analyses were performed by unpaired t-test with Prism (****p < 0.0001).*λ*_ex_ = 637 nm, *λ*_em_ = 685 – 735 nm. (C) Representative Flow cytometry analysis of apoptosis in CAL33 cells treated with BGT226, cetuximab and PAC-XL for 72 h. Q1: necrosis cells; Q2: late-stage apoptotic cells; Q3: early-stage apoptotic cells; Q4: live cells. Data are shown as mean ± SD (n = 3).

To define the functional consequences of PAC-XL, we examined cell-cycle progression, autophagy, and apoptosis. In CAL33 cells, PAC-XL (0.50–50 nM) increased the G0/G1 fraction from ~50% to ~75%, consistent with growth factor pathway suppression and similar to effects reported for cetuximab and BGT226 (Extended Data Fig. 8a). As a functional readout of mTOR inhibition, PAC-XL induced dose-dependent autophagy, increasing autophagic cells from 3.6% to 30% across 0–1000 nM. Consistent with a role for the payload, cetuximab did not induce detectable autophagy, whereas BGT226 did (Extended Data Fig. 8b). PAC-XL also triggered substantially more apoptosis than cetuximab (e.g., 53% vs. 12% early apoptosis at 100 nM; Extended Data Fig. 8c), similar to the response with free BGT226. Together, these results support a model in which PAC-XL enhances cytotoxicity primarily through targeted suppression of AKT–mTOR signaling, leading to G0/G1 arrest, autophagy induction, and increased apoptotic cell death.

**Extended Data Fig. 9 ∣:**
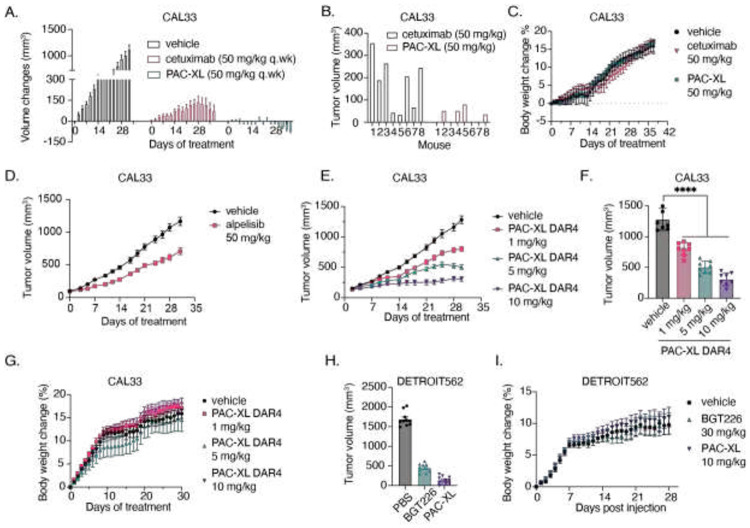
*In vivo* efficacy study of PAC-XL. (A) Tumor volume changes, (B) tumor volumes measured on the last day and (C) corresponding body weight changes in CAL33 xenograft-bearing mice treated with PBS vehicle, cetuximab or PAC-XL at 50 mg/kg, administered i.p. once weekly for 4 weeks. Data points are displayed as the mean ± SEM (n = 8). (D) Tumor growth curves in CAL33 xenograft-bearing mice treated with vehicle (1% methyl cellulose and 0.5% Tween 80 in water) or alpelisib at 50 mg/kg, administered i.p. once daily for 4 weeks. (E) Tumor growth curves, (F) tumor volumes measured on the last day and (G) corresponding body weight changes in CAL33 xenograft-bearing mice treated with PBS vehicle, cetuximab or PAC-XL (DAR = 4) at 1, 5 and 10 mg/kg, administered i.p. twice weekly for 4 weeks. Data points are displayed as the mean ± SEM (n = 8). (H) tumor volumes measured on the last day and (I) corresponding body weight changes in DETROIT562 xenograft-bearing mice. BGT226 was administered i.p. once daily at 30 mg/kg; PBS vehicle and PAC-XL were administered i.p. twice weekly at 10 mg/kg. Data points are displayed as the mean ± SEM (n = 10). Statistical analysis of final tumor volumes was performed across all groups using one-way ANOVA in Prism. ****p < 0.0001.

After establishing selective *in vitro* activity, we next evaluated PAC-XL in a CAL33 xenograft model. Cetuximab and PAC-XL were administered intraperitoneally (i.p.) at 1, 5, and 10 mg/kg twice weekly for four consecutive weeks, and tumor volumes were measured three times per week. Compared with vehicle control, PAC-XL induced robust and sustained tumor growth inhibition and was markedly more effective than dose-matched cetuximab (Fig. 2e, 2f). Body weight remained stable across all treatment groups (Fig. 2g), indicating good tolerability. Notably, PAC-XL achieved complete tumor growth suppression at both 10 mg/kg twice-weekly and 50 mg/kg once-weekly dosing, with 4 of 8 mice exhibiting complete tumor regression after four weeks of treatment (Fig. 2f; Extended Data Fig. 9a, 9b). Consistently, PAC-XL was well tolerated and did not induce significant body weight loss even at the highest dose tested (Extended Data Fig. 9c). Furthermore, even at a low dose (1 mg/kg, twice weekly, Fig. 2e), PAC-XL (TGI=62%) outperformed the clinically approved PI3Kα inhibitor alpelisib (50 mg/kg, daily; Extended Data Fig. 9d, TGI=42%), highlighting its strong *in vivo* efficacy. As many existing ADCs show optimal activity at DAR ~4, we evaluated PAC-XL at DAR 4 *in vivo.* As shown in Extended Data Fig. 9e-g, DAR 4 PAC-XL, dosed at 1, 5, and 10 mg/kg twice weekly, showed lower antitumor activity than the DAR 8 construct. Notably, 5 mg/kg of DAR 4 PAC-XL achieved efficacy similar to 1 mg/kg of DAR 8, highlighting the greater potency of the higher-DAR construct. These results suggest that optimized labeling chemistry can enable increased drug loading while maintaining or improving therapeutic efficacy. We further used an HNSCC cell-line, DETROIT562, xenograft tumor–bearing mice to compare the efficacy of PAC-XL with that of the free BGT226 payload.^16^ PAC-XL (b.i.w., i.p., 10 mg/kg) led to superior tumor growth inhibition (Fig. 2h) compared to BGT226 (q.d., 30 mg/kg) with no impact on body weight (Extended Data Fig. 9i).

**Extended Data Fig. 10 ∣:**
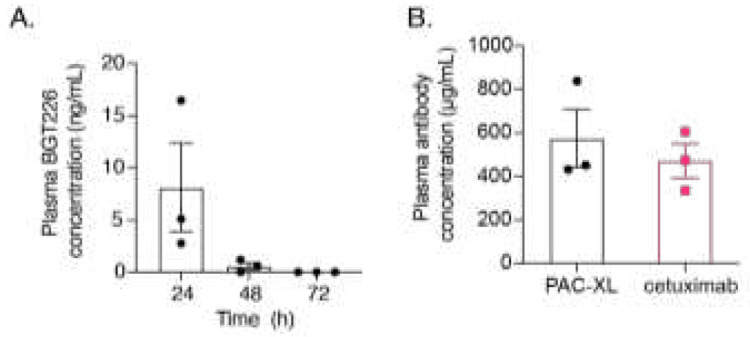
Pharmacokinetic study of PAC-XL. (A) HPLC–QTOF–MS analysis of BGT226 concentration in CD-1 mice plasma after intraperitoneal treatment with 100 mg/kg PAC-XL over 72 hours. (B) Total antibody concentration measured by ELISA in CD-1 mouse plasma 72 hours after intraperitoneal administration of PAC-XL (100 mg/kg) or cetuximab (100 mg/kg). Data are presented as mean ± SEM (n = 3).

Because dose-limiting toxicities constrain systemic PI3K–mTOR inhibitors, we compared acute metabolic and tolerability signals in CD-1 mice. Alpelisib (50 mg/kg) induced rapid hyperglycemia that persisted beyond 4 h (Fig. 2i).^30^ In contrast, neither PAC-XL nor cetuximab (100 mg/kg) produced significant blood glucose elevation. Although BGT226 did not cause marked hyperglycemia in our conditions, it has been associated with substantial systemic adverse effects in clinical testing, including gastrointestinal toxicity, fatigue, and hepatotoxicity.^19^ In this study, BGT226 (50 mg/kg) caused pronounced body-weight loss, while PAC-XL caused no measurable weight reduction (Fig. 2J). An initial pharmacokinetic (PK) study indicated the plasma BGT226 concentration was below 20 ng/mL after 24 h of treatment with 100 mg/kg PAC-XL (Extended Data Fig. 10a). By contrast, the plasm antibody concentration, as assessed by an ELISA, for PAC-XL was ~0.5 mg/mL, similar to that of cetuximab (Extended Data Fig. 10b). These studies lend support to the therapeutic potential of PAC-XL.

This study introduces precision antibody conjugates (PACs) as a strategy for treating HNSCC, combining antibody targeting with delivery of pathway-selective inhibitors. PAC-XL couples cetuximab to BGT226, a potent dual PI3K/mTOR inhibitor that showed strong preclinical activity but was limited clinically by systemic toxicity. Central to this approach is the use of new linker technology as a standard Val–Cit PAB linker led to aggregation and low DAR or, when PEGylated, loss of functional activity consistent with inefficient payload release. Using a β-glucuronidase-cleavable benzyl-ammonium carbamate (BAC) linker, we generated a homogeneous, high-DAR conjugate, PAC-XL, that emerged as a promising compound across a series of characterization efforts. Enabled by imaging-driven assays, PAC-XL, displayed preserved EGFR binding, remained stable in plasma, and released payload only after trigger activation.

Clinically, PAC-XL addresses two linked problems: EGFR blockade alone is rarely durable, and PI3K/AKT/mTOR activation is ubiquitous in HNSCC and contributes to both cancer cell survival and resistance. By concentrating PI3K/mTOR inhibition in EGFR-expressing tumors, PAC-XL produced potent cytotoxicity and pathway suppression in PIK3CA-altered, EGFR-dependent HNSCC models and diminished activity in EGFR- or PI3K-independent lines. These findings suggest this approach as a route to combine receptor targeting with orthogonal pathway blockade, especially in settings where systemic inhibitor dosing is constrained clinically. Our studies disclosing PAC-XL have broader implications for a more global strategy to combine precision therapies with antibodies and likely can be applied to numerous small molecule signaling inhibitors including KRAS and RAF/MEK/ERK pathway inhibitors. Going forward, given that immune checkpoint blockade is central in recurrent/metastatic HNSCC, PAC-XL is an attractive partner for anti–PD-1 therapy. mTOR suppression has been shown to complement immunotherapy, potentially reshaping antigen availability and inflammatory signaling within the tumor microenvironment.^31^ Targeted PI3K/mTOR inhibition may reshape tumor metabolism, antigen presentation, and inflammatory signaling in a tumor targeted fashion, but full evaluation will require immunocompetent models and more likely, clinical characterization.

Nevertheless, several key translational questions remain. Clinical efficacy will likely depend on analysis of EGFR and PI3K pathway dependence. Resistance could arise through EGFR downregulation or signaling via alternative pathways. Additionally, our data and others suggest that the β-glucuronide linker may be cleaved both intra- and extra-cellularly. While this property could improve efficacy in settings of variable antigen expression, it will require careful evaluation. Overall, these studies support PACs as a practical strategy to repurpose potent but previously intolerable signaling inhibitors, and more broadly, this work illustrates how linker innovation can expand the payload repertoire beyond conventional cytotoxic agents.

## Methods

### Synthesis

Detailed chemical synthesis and characterization of new compounds can be found in Supplementary Information.

### Reagents and Apparatus

Unless otherwise stated, all reagents, including metal ions, thiols, H_2_O_2_, and other chemicals, were purchased from Sigma-Aldrich, VWR, Oakwood, Ambeed, or AA Blocks at a high commercial quality and used without further purification. γ-Glutamyltranspeptidase from equine kidney (Lot#101M7028), nitroreductase from *Escherichia coli* (Lot # 0000469026), cathepsin B from human liver (Lot # SLCG5308), elastase from porcine pancreas (Lot # 0000327494), β-glucuronidase (type IX-A) from *Escherichia coli* (Lot # 0000261827), β-galactosidase from *Escherichia coli* (Lot # 0000419984), hydrocortisone, mouse plasma and human plasma were ordered from Sigma-Aldrich. BGT226 was obtained from MedChemExpress. Cetuximab and rituximab were ordered from the Division of Veterinary Resources Pharmacy. Phosphate buffered saline (PBS, 10 mM, pH 7.2), HEPES buffer (1M, pH 7.3), LysoTracker^™^ Green FM (Lyso Green), NucBlue^™^ Live Cell Stain Ready Probes^™^ reagent (Hoechst 33342), Annexin V-FITC conjugate, propidium iodide, RNase A, Dulbecco’s Modified Eagle’s medium (DMEM), Minimum Essential medium (MEM), RPMI 1640 medium, McCoy's 5A (modified) medium, fetal bovine serum (FBS), penicillin-streptomycin (pen-strep), trypsin/EDTA solution, cell dissociation buffer, and MEM non-essential amino acids solution (NEAA) were ordered from ThermoFisher Scientific. Autophagy Red assay was ordered from ImmunoChemistry Technologies, LLC. BIAcore CM5 chips were obtained from Cytiva. Human epidermal growth factor receptor (EGFR) was purchased from ACRO Biosystems. Ultrapure water (over 18 MΩ·cm) produced by a Milli-Q reference system (Millipore) was used throughout the whole experiments.

Flash column chromatography was performed using Redisep Silver silica gel columns (60 Å, 40-60-micron particle size) and Biotage Sfär C18 Duo Columns (100 Å, 30-micron particle size) on a CombiFlash Rf 200i (Teledyne Isco, Inc.). Fast protein liquid chromatography was conducted on Cytiva ÄKTA pure protein purification system with a Superdex 200 Increase 10/300 GL size-exclusion column and a detection wavelength of 280 nm. ^1^H NMR spectra (at 400 or 500 MHz) and ^13^C NMR spectra (100 or 125 MHz) were recorded on Bruker spectrometers in Methanol-*d_4_*, CDCl_3_, CD_3_CN or D_2_O (Cambridge Isotope Laboratories). Chemical shifts are presented in ppm and referenced by residual solvent peak. High-resolution LC/MS analyses were conducted on a 6250 Accurate-Mass Quadrupole Time-of-Flight (Q-TOF) LC/MS (Agilent Technologies, Inc.). Small molecules were injected onto a Poroshell 300SB-C18 column (2.1 × 75 mm), and eluted with 5% – 100% CH_3_CN/H_2_O gradient with 0.1% aqueous formic acid over 5 mins (flow rate = 1.0 mL/min). Antibody conjugates were analyzed by a Poroshell 300SB-C3 column (2.1 × 75 mm) and the elution employed 5% – 100% CH_3_CN/H_2_O gradient with 0.1% aqueous formic acid over 35 mins (flow rate = 0.25 mL/min). The spectral data were collected using MassHunter Workstation and deconvoluted with Agilent MassHunter Bioconfirm software. Absorbance curves were obtained on a Jasco V-770 spectrophotometer. Fluorescence spectra were measured on a Horiba fluorimeter QM-8075-11-C, with 5 nm excitation and emission slit widths, and a 0.1 s integration rate. Analytical LC analyses were collected from Agilent 1260 Infinity Quaternary LC module using Poroshell 120 EC-C18 2.7 μM (4.6 × 50 mm) column in 5% – 95% CH_3_CN/H_2_O gradient with 0.1% formic acid over 25 minutes (flow rate = 1.0 mL/min). Hydrophobic interaction chromatography was performed on an Agilent 1260 series HPLC with the MabPac^™^ HIC-10 column (4.6 mm × 100 mm). Surface plasmon resonance (SPR) analysis was performed on a Biacore 3000 SPR system, and data was processed with the BIAEvaluation 4.0 software. MTS [3-(4,5-dimethylthiazol-2-yl)-5-(3-carboxymethoxyphenyl)-2-(4-sulfophenyl)-2H-tetrazolium] analyses and alamarBlue assays were conducted on a Synergy Mx microplate reader. Confocal fluorescence images were performed on an Andor spinning disk confocal on Leica DMi8 base and image processing was carried out with Fiji. Flow cytometry was carried out using a BD FACSymphony A5 cell analyzer. The data was analyzed by FlowJo 10.7.1 software.

### Generation and Purification of Antibody Conjugates Using NHS-Lysine Labeling

In 1.5 mL Eppendorf tubes, 1 mL of antibodies (2 mg/mL) were mixed with 10 equivalents of NHS esters (13.6 μL of 10 mM DMSO stock solution) in PBS (10 mM, pH 7.2) and gently shaken at room temperature for 2 h. The resulting solutions were first purified by PD-10 columns with PBS (10 mM, pH 7.2), following by fast protein liquid chromatography (FPLC), eluted with PBS at a flow rate of 0.75 mL/min. UV absorbance was detected at 280 nm. The monodispersed conjugate fractions were collected, concentrated using centrifugal filter (10 kDa cutoff) and stored at 4 °C. All conjugates were used for animal studies within a week after purification.

### Generation and Purification of Antibody Conjugates Using Maleimide-Cysteine Labeling

In 5.0 mL Eppendorf tubes, antibodies (2 mL, 2 mg/mL in 10 mM PBS, pH 7.2) were reduced with 40 equivalents of tris(2-carboxyethyl)phosphine (TCEP; 22 μL of a 50 mM aqueous stock solution). The reaction mixtures were incubated at 37 °C for 1 h. Excess TCEP was subsequently removed by dialysis using Slide-A-Lyzer^™^ Mini dialysis devices (10 kDa cutoff) against PBS (10 mM, pH 7.2) containing 1.0 mM EDTA at 4 °C for 24 h. The reduced antibody solutions were then transferred to fresh 5.0 mL Eppendorf tubes and reacted with 10 equivalents of maleimide-bearing payloads (27 μL of 10 mM stock solutions in DMSO). The reaction mixtures were gently agitated at room temperature for 3 h. The crude conjugates were first purified by PD-10 desalting columns equilibrated with PBS (10 mM, pH 7.2), followed by FPLC using PBS as the mobile phase at a flow rate of 0.75 mL/min. Elution was monitored by UV absorbance at 280 nm. The monodispersed conjugate fractions were collected, concentrated using centrifugal filters (10 kDa cutoff), and stored at 4 °C. All conjugates were used for animal studies within a week after purification.

### Drug Antibody Ratio Calculation with Absorbance

The drug antibody ratio (DAR) of Cet-VCB, Cet-PVCB, and PAC-XL was determined by the following equation.


$$DAR=\frac{A_{340}\varepsilon_{antibody}}{(A_{280}-A_{340}C_{280})\varepsilon_{payload}}$$

A_340_ is the absorption value of the conjugate at 340 nm, A_280_ is the absorption value of the conjugate at 280 nm, and $\varepsilon_{\text{antibody}}$ is 210,000 M^−1^cm^−1^. A correction for each payload at 280 nm (C_280_) was applied to account for the absorption contribution of the payload at 280 nm relative to 340 nm, which was determined by A_280_/A_340_ of a free payload absorbance in PBS. The payloads molar extinction coefficients ($\varepsilon_{\text{payload}}$) and correction factors (C_280_) are listed below.

**Table:** 

	BGT-VC-NHS	BGT-PVC-Mal	BGT-BAC-Glu-Mal
$\varepsilon_{\text{payload}}$ (M^−1^ cm^−1^)	9860	9860	6290
C_280_	1.69	1.69	4.2

The degree of labeling (DOL) of Cetux-FNIR766, Ritux-FNIR766 and Cet-Hcy-GluBAC was determined by the following equation.


$$DOL=\frac{A_{payload}\varepsilon_{antibody}}{(A_{280}-A_{payload}C_{280})\varepsilon_{payload}}$$

$A_{\text{payload}}$ is the absorbance of the conjugate measured at the wavelength corresponding to the maximum absorbance of the payload (766 nm for Cetux-FNIR766, Ritux-FNIR766, and 588 nm Cet-Hcy-GluBAC), A_280_ is the absorption value of the conjugate at 280 nm, and $\varepsilon_{\text{antibody}}$ is 210,000 M^−1^cm^−1^. A correction for each payload at 280 nm (C_280_) was applied to account for the absorption contribution of the payload at 280 nm relative to the absorbance at the dye’s maximum wavelength which was determined by A_280_/A_766_ or A_280_/A_588_ of a free payload absorbance in PBS. The payloads molar extinction coefficients ($\varepsilon_{\text{payload}}$) and correction factors (C_280_) are listed below.

**Table:** 

	FNIR766-NHS	Hcy-BAC-Glu-Mal
$\varepsilon_{\text{payload}}$ (M^−1^ cm^−1^)	188100	48800
C_280_	0.06	0.16

The DOL of PAC-XL-FNIR766 was determined by the following set of equations.


$$A_{280}=\varepsilon_{cetux}\times C+\varepsilon_{drug}\times C_{280-drug}\times DAR_{drug}\times C+\varepsilon_{dye}\times C_{280-dye}\times DOL_{dye}\times C\;\;A_{340}=\varepsilon_{drug}\times DAR_{drug}\times C+\varepsilon_{dye}\times C_{340-dye}\times DOL_{dye}\times C\;\;A_{766}=\varepsilon_{dye}\times DOL_{dye}\times C$$

A_280_ and A_340_ are the absorbance of PAC-XL-FNIR766 at 280 nm and 340 nm. C is the concentration of the measured conjugate; $\varepsilon_{\text{cetux}}$, $\varepsilon_{\text{drug}}$ and $\varepsilon_{\text{dye}}$ is the molar extinction coefficient of cetuximab (210,000 M^−1^cm^−1^), BGT-BAC-Glu-Mal (6290 M^−1^cm^−1^) and FNIR766-NHS (188,100 M^−1^cm^−1^). DAR_drug_ is the drug antibody ratio for payload BGT-BAC-Glu-Mal, which is determined as 8.0. DOL_dye_ is the degree of labeling for payload FNIR766-NHS. C_280-drug_ is the correction factor for the BGT-BAC-Glu-Mal payload at 280 nm, which was determined to be 4.2 as shown above. C_280-dye_ is the correction factor for the FNIR766-NHS payload at 280 nm, which was determined to be 0.06 as described above. C_340-dye_ is the correction factor of the FNIR766-NHS payload at 340 nm. This value was calculated as the ratio of the absorbance of the free dye at 340 nm to its absorbance at the dye’s maximum wavelength (766 nm) in PBS, yielding a correction factor of 0.0297.

### Drug Antibody Ratio Calculation with HPLC–QTOF–MS

The DAR of PAC-XL was also analyzed by Agilent 6250 Accurate-Mass Quadrupole Time-of-Flight LC/MS (HPLC–QTOF–MS) equipped with a dual electro-spray source, operated in the positive-ion mode. Antibody conjugates were first reduced with 40 eq of TCEP at room temperature for 30 min and then diluted with 10-fold water before subjecting to HPLC–QTOF–MS equipped with a Poroshell 300SB-C3 column (2.1 × 75 mm, particle size 5 μm). The analytes were eluted at a flow rate of 0.25 mL/min with a 5 to 100% organic gradient over 35 minutes. Both mobile phases, water and acetonitrile, contained 0.1% formic acid. The instrument was used in a full-scan TOF mode. MS source parameters were set with a capillary voltage of 4 kV, the fragmentor voltage of 300 V and skimmer 65 V. The gas temperature was 350 °C, drying gas flow 12 l/min and nebulizer pressure 55 psig. Data were acquired at high resolution (3,200 *m/z*), 4 GHz. TOF-MS mass spectra were recorded across the range 100–3,200 *m*/z. To maintain mass accuracy during the run time, an internal mass calibration sample was infused continuously during the LC/MS runs. Data acquisition was performed using Mass Hunter Workstation (version B.06.01). For data analysis and deconvolution of mass spectra Mass Hunter Qualitative Analysis software (version B.07.00) with Bioconfirm Workflow was used.

The DAR of PAC-XL (${\text{DAR}}_{\text{total}}$) was determined by comparing the deconvoluted molecular masses of the heavy and light chains ($M_{\text{conjugated}\;\text{chain}}$) with those of unmodified cetuximab ($M_{\text{native}\;\text{chain}}$). The average mass increase per chain, relative to the molecular mass of the payload ($M_{\text{payload}}$), was calculated to determine ${\text{DAR}}_{\text{total}}$. The equations for DAR calculation are shown below.


$$\;DAR_{chain}=\frac{M_{conjugated\;chain}-M_{native\;chain}}{M_{payload}}\;DAR_{total}=2\times (DAR_{heavy\;chain}+DAR_{light\;chain})$$

### Hydrophobic Interaction Chromatography Analysis

To compare the hydrophobicity of PAC-XL and cetuximab, hydrophobic interaction chromatography (HIC) was performed using an Agilent 1260 series HPLC system equipped with a MabPac^™^ HIC-10 column (4.6 mm × 100 mm). Conjugates (2.0 mg/mL, 20 μL injection volume) were loaded onto the column at a flow rate of 1.0 mL/min. Mobile phase A consisted of 1.5 M ammonium sulfate in 25 mM Tris-HCl (pH 8.0), while mobile phase B consisted of 25 mM Tris-HCl (pH 8.0) containing 5% isopropanol (v/v). A linear gradient from 0% to 100% mobile phase B was applied over 20 min. Elution was monitored by measuring absorbance at 280 nm.

### SPR Assay

BIAcore CM5 chip coated with carboxylated dextran was utilized for target protein (human-recombinant EGFR) immobilization. The chip was equilibrated with HBS-EP buffer (10 mM HEPES, 150 mM NaCl, 3 mM EDTA, and 0.005% Tween-20) at a flow rate of 5 μL/min, followed by injecting activation solution (0.1 M NHS and 0.4 M EDC) into Fc2 for 15 min. EGFR was passed over the activated surface at a concentration of 10 μg/mL in 10 mM sodium acetate (pH 4.5) for 20 min, resulting in an immobilization amount of 4600 RU. Then, 1 M ethanolamine (pH 8.5) was applied to block the residual NHS-ester. After quenching, 10 mM NaOH regeneration buffer was injected as a pulse (1 min) to remove unbound proteins. The immobilization was considered complete when the baseline was stable after regeneration. Fc 1 remained blank as a reference channel during the whole procedure.

The binding affinity of cetuximab and PAC-XL to EGFR was then measured at room temperature. PBS was used as the running buffer flowing through Fc1 (reference channel) and Fc2 (EGFR channel) at 25 μL/min, and 10 mM NaOH was used for regeneration of the chip surface. For affinity determination, a serial dilution (1:1 ratio) of the antibody solution was injected starting from 200 nM. For each concentration of cetuximab and PAC-XL, 180 s association and 300 s dissociation were applied. The equilibrium dissociation constant (K_D_) and kinetics (k_a_ and k_d_) were obtained to characterize the binding by using the BIAEvaluation 4.0 software with the Langmuir 1:1 binding model.

### Small Molecular Characterization with HPLC–QTOF–MS

Mass spectrometry data were acquired on an Agilent 6520 Accurate-Mass Quadrupole Time-of-Flight LC/MS System (HPLC–QTOF–MS), equipped with a dual electro-spray source, operated in the positive-ion mode. Separation was performed on 300SB-C18 Poroshell column (2.1 mm × 75 mm; particle size 5 μm). The analytes were eluted at a flow rate of 1.0 mL/min with a 5 to 100% organic gradient over 4 minutes and holding organic for 1 minute, where 0.1% aqueous formic acid was exchanged for 0.1% formic acid in acetonitrile. The instrument was used in either full-scan TOF mode. MS source parameters were set with a capillary voltage of 4 kV, the fragmentor voltage of 150 V and skimmer 65 V. The gas temperature was 350 °C, drying gas flow 12 l/min and nebulizer pressure 55 psig. Data were acquired at high resolution (1,700 *m/z*), 4 GHz. To maintain mass accuracy during the run time, an internal mass calibration sample was infused continuously during the LC/MS runs. Data acquisition and analysis were performed using MassHunter Workstation Data Softwares, LCMS Data Acquisition (version B.06.01) and Qualitative Analysis (version B.07.00).

### Stability Assessment Assay

To test the stability of PAC-XL under various biological triggers, 5.0 μM (payload concentration) of PAC-XL were mixed with either pH 4.0 PBS (100 mM), or pH 8.0 PBS (100 mM), or with a series of biologically relevant species, including nitroreductase (20 U/mL), cathepsin B (5 U/mL), elastase (1 U/mL), β-glucuronidase (5 KU/mL), β-galactosidase (5 U/mL), GGT (*γ*-glutamyltranspeptidase, 10 U/mL), GSH (glutathione, 10 mM), cysteine (100 μM), Na_2_S (100 μM), and H_2_O_2_ (100 μM). All bio species were tested in PBS (10 mM, pH 7.2) except from cathepsin B, which was performed in acetate sodium buffer (50 mM, pH 5.0) with 1.0 mM TCEP and 1.0 mM EDTA. After incubation at 37 °C for 24 h in a thermostat, a 50-μL portion of each reaction solution was collected and mixed with same volume of acetonitrile. The mixture was centrifuged at 15,000 rpm for 10 min to remove the precipitates, and the supernatant was subjected to HPLC–QTOF–MS and characterized with the small molecular characterization method described above. The percentage of released BGT226 was then evaluated based on a standard curve of BGT226 (shown below) in PBS (10 mM, pH 7.2) and acetonitrile (1:1, v/v).


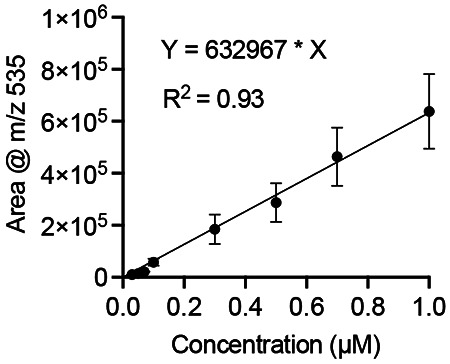


To test the stability of PAC-XL in human and mouse plasma, PAC-XL (25 μL, 0.8 mg/mL) was added to 175 μL of 2-fold diluted human plasma or complete mouse plasma to prepare the working solution. Aliquots (50 μL, 100 μg/mL) from the working solution were incubated at 37 °C. Each sample was collected at set time points and frozen at −80 °C. After collecting the final aliquots, all samples were defrosted at room temperature, diluted with 50 μL of acetonitrile and centrifuged at 15,000 rpm for 10 min to remove proteins. The supernatant was subjected to HPLC–QTOF–MS and characterized with the small molecular characterization method described above. The percentage of released BGT226 was then evaluated based on the standard curve of BGT226 (shown above).

To test the Retro-Michael deconjugation of the thiosuccinimide linkage, 5.0 μM of PAC-XL (payload concentration) was incubated with 0.50 mM of N-acetylcysteine (NAC) in PBS (10 mM, pH 7.2) at 37 °C. Aliquots (50 μL, 100 μg/mL) were collected at set time points and frozen at −80 °C. After collecting the final aliquots, all samples were defrosted at room temperature and diluted with 50 μL of acetonitrile. The supernatant was subjected to HPLC–QTOF–MS and characterized with the small molecular characterization method described above. The percentage of released BGT226 was then evaluated based on the standard curve of NAC-BGT (shown below) acquired in PBS (10 mM, pH 7.2) and acetonitrile (1:1, v/v).


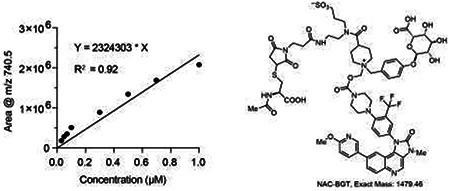


To evaluate the extracellular cleavage of PAC-XL, cells were seeded in 96-well plate (1,000 cells/well) and cultured for 5 days under standard incubation conditions. The culture media were then collected and centrifuged at 400 g for 6 min. The supernatants were transferred to new tubes, and PAC-XL (0.1 mg/mL) was added to either the conditioned media or fresh media (control group), followed by incubation at 37 °C for 5 days. At set time points, 50 μL aliquots were collected and stored at −80 °C. After the final time point was collected, all samples were thawed at room temperature and mixed with an equal volume (50 μL) of acetonitrile. The mixtures were centrifuged at 15,000 rpm for 10 min, and the resulting supernatants were analyzed by HPLC–QTOF–MS and characterized with the small molecular method described previously. The percentage of released BGT226 was quantified based on a calibration curve of BGT226 prepared in PBS (10 mM, pH 7.2) and acetonitrile (1:1, v/v) as shown above.

### Cell Culture

CAL33 (DSMZ, October 2012), JIMT1(ATCC, November 2010) cells were maintained in DMEM supplemented with 10% FBS, 1% pen-strep. BICR56 (Public Health England, April 2014) cells were maintained in DMEM supplemented with 10% FBS, 1% pen-strep, and 0.4 μg/mL hydrocortisone, DETROIT562 (ATCC, November 2014) and MCF7 (ATCC, November 2010) cells were maintained in MEM with 10% FBS and 1% pen-strep. HCC1428 (ATCC, May 2025), DLD1, and BEAS2B (ATCC, October 2013) cells were maintained in RPMI 1640 with 10% FBS and 1% pen-strep. RPMI2650 (ATCC, May 2025) cells were maintained in MEM with 10% FBS, 1% pen-strep and 1% NEAA. HCT116 cells (ATCC, October 2013) were maintained in McCoy’s 5A with 10% FBS, 1% pen-strep. Cells were grown in a humidified 5% CO_2_ incubator at 37 °C, and passaged when confluency reached 80%. Cells were evaluated for molecular testing of biological materials by animal health diagnostic laboratory at Frederick National Laboratory for Cancer Research. The results confirmed the absence of the following agents within the cells: Ectromelia virus (ECT), Mouse rotavirus (EDIM), Lymphocytic coriomeningitis virus (LCMV), Lactic dehydrogenase elevating virus (LDHV), Mouse adenovirus (MAD), Mouse cytomegalovirus (MCMV), Mouse hepatitis virus (MHV), Mouse norovirus (MNV), Mouse parvovirus (MPV), Minute virus of mice (MVM), Mycoplasma spp. (MYCO), Polyoma virus (POLY), Pneumonia virus of mice (PVM), Reovirus 3 (REO3), Sendai virus (SEN), Theiler's murine encephalomyelitis virus (TMEV).

### Intracellular Fluorescence Imaging

Confocal imaging was carried out at Optical Microscopy and Analysis Core (OMAC). CAL33 cells were seeded in 10-well cellview slides (Greiner bio-one) at a density of 40,000 cells/well for 24 h to adhere before experiments. For imaging, the cells were incubated with 200 μL of PAC-XL-FNIR766 (5.0 nM, FNIR766 concentration) at 4 °C for 4 h or 37 °C for 18 h. Before imaging, cells were further co-incubated with Lyso Green (100 nM) and 20 μL of commercial Hoechst 33342 solution for 10 min at 4°C or 37 °C. The images were taken at 63×/1.4 water immersed objective with an excitation of 730 nm, 488 nm, and 405 nm for PAC-XL-FNIR766, Lyso Green and Hoechst 33342, respectively.

### Flow Cytometry Analysis

Flow cytometry was performed at the NCI-Frederick CCR Flow Cytometry Core Facility to quantify cellular binding of PAC-XL, assess intracellular payload release, and elucidate the mechanism of action of PAC-XL, including its effects on cell cycle distribution, autophagy, and apoptosis. Gating strategy is shown in Supplementary Informention. Each experiment was performed in triplicate and 10,000 cells were counted in each analysis.

To quantify the binding of PAC-XL in cells with different expression levels of EGFR, cells were plated into 12-well plate (300,000 cells/well) and incubated with PAC-XL-FNIR766, Cetux-FNIR766, or Ritux-FNIR766 (20 nM, FNIR766 concentration) at 37 °C in complete media for 24 h. The media was then removed and cells were washed with PBS (600 μL × 2/well). Cell dissociation buffer (400 μL/well) was added and incubated with cells for 15 min in 37 °C incubator. Subsequently, the dissociation buffer was discarded, and cells were suspended in 200 uL of ice-cold PBS (10 mM, pH 7.2). The cell suspension was transferred to flow cytometer tubes and stored on ice bath under dark until the flow cytometry experiment. The fluorescence signal was excited using a 637 nm laser and emission was detected on APC-Cy7 detectors (780/60). Geometric mean fluorescent intensity was calculated in APC-Cy7 channel.

To evaluate the intracellular cleavability of PAC-XL, CAL33 cells were seeded in 12-well plates (300,000 cells/well) and allowed to adhere overnight. Cells were pretreated with the β-glucuronidase inhibitor baicalin (0 – 500 μM) for 1 h, followed by incubation with the fluorogenic PAC-XL analogue Cet-Hcy-GluBAC (50 μg/mL) in complete media at 37 °C for 24 h. The media was then removed and cells were washed with PBS (600 μL × 2/well). Cell dissociation buffer (400 μL/well) was added and incubated with cells for 15 min in 37 °C incubator. Subsequently, the dissociation buffer was discarded, and cells were suspended in 200 uL of ice-cold PBS (10 mM, pH 7.2). The cell suspension was transferred to flow cytometer tubes and stored on ice bath under dark until the flow cytometry experiment. The fluorescence signal was excited using a 637 nm laser and emission was detected on Alexa Fluor 700 detectors (710/50). Geometric mean fluorescent intensity was calculated in Alexa Fluor 700 channel.

To evaluate the cell cycle arrest of PAC-XL, CAL33 cells (200,000 cells/well) were seeded in 6-well plates for 1 day and then incubated with PAC-XL, cetuximab or BGT226 at various concentrations (0 – 50 nM) at 37 °C for 2 days. All cells of each well were harvested into 1.5 mL tubes and fixed first with 5% paraformaldehyde at 4 °C for 30 min and then with 70% cold ethanol at −20 °C for 2 h. After being washed twice with cold PBS, these cells were stained with 50 μg/mL propidium iodide solution containing 50 μg/mL RNase A and 0.1% Triton-X100 for 30 min at 37 °C in the dark before being analyzed by flow cytometry. The fluorescence signal was excited using a 561 nm laser and emission was detected on PE-CF594 detectors (610/20). Cell cycle analysis was performed using Watson model.

For the autophagy analysis, CAL33 cells (200,000 cells/well) were seeded in 6-well plates for 1 day and then incubated with PAC-XL, cetuximab or BGT226 at various concentrations at 37 °C for 3 days. All cells of each well were harvested into 1.5 mL tubes. After being washed once with cold PBS, these cells were stained with Autophagy Red for 60 min at 37 °C before being analyzed on flow cytometry. The fluorescence signal was excited using a 637 nm laser and emission was detected on Alexa Fluor 700 detectors (710/50). Geometric mean fluorescent intensity was calculated in Alexa Fluor 700 channel.

For the apoptosis analysis, CAL33 cells (200,000 cells/well) were seeded in 6-well plates for 1 day and then incubated with PAC-XL, cetuximab or BGT226 at various concentrations at 37 °C for 3 days. All cells of each well were harvested into 1.5 mL tubes. After being washed once with cold PBS, these cells were stained with Annexin V-FITC and propidium iodide solution for 15 min at room temperature in the dark before being analyzed on flow cytometry. Fluorescence signals were excited using a 488 nm laser for FITC and a 561 nm laser for propidium iodide, and emission was detected using BB515 (515/20) and PE-CF594 (610/20) detectors, respectively.

### *In Vitro* Cell Viability Assay

The cytotoxicity of Cet-VCB and Cet-PVCB conjugates to CAL33 cells was examined by standard MTS assay. Briefly, cells were seeded in 96-well plates (3,000 cells/well) and incubated at 37 °C for 24 h at which time, the cells were treated with the corresponding drugs and concentrations in complete cell culture media. After 72 h, 15 μL of premade MTS reagent was added and the plates were incubated at 37 °C for at least 1.5 h. The absorbances were taken at 590 nm with a microplate reader. The cell viability rate (VR) was calculated according to the equation: $\text{VR}=A∕A_{0}\times 100\%$, where A is the absorbance of the experimental group (i.e., the cells were treated by conjugates) and A0 is the absorbance of the control group (i.e., the group treated with culture media only). The cell survival rate from the control group was considered to be 100%. The data was analyzed in GraphPad Prism10 using the non-linear regression (four-parameter) algorithm.

The cytotoxicity of PAC-XL was examined by alamarBlue assay. Briefly, cells were seeded in 96-well black side plates (1,000 cells/well) at 37 °C for 24 h at which time, cells were treated with the corresponding drugs and concentrations in complete cell culture media. Each concentration was repeated for 8 times in one plate. After 5 days, the culture media were discarded, and 100 μL of the alamarBlue solution (10%, v/v) was added to each well, followed by incubation at 37 °C for 4 h. After shaking the plates for 10 min, fluorescence values of the wells were collected with a microplate reader at 590 nm under an excitation of 560 nm. For each concentration, three of the eight wells were used to calculate cell viability, and these were considered one experimental replicate. The experiment was performed in triplicate. The cell viability rate (VR) was calculated according to the equation: $\text{VR}=\Delta F∕\Delta F_{0}\times 100\%$, where ΔF is the fluorescence of the experimental group deducts the fluorescence of the blank wells (i.e., wells without any cells), and F_0_ is the fluorescence of the control (i.e., wells treated with culture media only) deducts the fluorescence of the blank wells. The cell survival rate from the control group was considered to be 100%. The data was analyzed in GraphPad Prism10 using the non-linear regression (four-parameter) algorithm.

### Western Blot Analysis

Cells were plated in 6-well plates (300,000 cells/well) and incubated at 37 °C for 24 h prior to treatment with the indicated drugs in complete media. After 24 h of treatment, cells were washed twice with ice-cold PBS (10 mM, pH 7.2) and lysed in RIPA buffer (Sigma-Aldrich), supplemented with protease inhibitor (Sigma-Aldrich), and phosphatase inhibitor cocktails 2 and 3 inhibitors (Sigma-Aldrich). A total of 150 μL lysis buffer was added per well, and cells were scraped, collected, and centrifuged at 15,000 rpm for 10 min at 4 °C. Protein concentrations were determined using Pierce’s 660 nm protein assay reagent (Thermo Scientific), and samples were normalized prior to denaturation to ensure equal protein loading. Samples were denatured and subjected to SDS-PAGE (Bio-Rad, 4 – 20% acrylamide gradient gels), and transferred to PVDF membranes (Bio-Rad) and blocked for 2 h using 5% bovine serum albumin (BSA) in tris-buffered saline supplemented with 0.1% Tween^®^20 (TBS-T) (Sigma-Aldrich) and 0.01% sodium azide (Sigma-Aldrich). Membranes were then incubated overnight at 4 °C with the following primary antibodies in 5% BSA/TBS-T (all at 1:1000 dilution from cell signaling technology): AKT (#2920S), pAKT S473 (#4060S), pPRAS40 T246 (#13175S), tPRAS40 (#2691S), p4EBP1 Thr37/46 (#2855T), t4EBP1 (#9644T), pP70 S6 (#9234T), tP70 S6 (#9202S), and GAPDH (#2118S). After washing, membranes were incubated with appropriate horseradish peroxidase-conjugated secondary antibodies (anti-mouse cat #NA931; and antirabbit #NA934; GE Healthcare Life Sciences) for 1 h at room temperature and protein bands were visualized using enhanced chemiluminescence detection (Bio-Rad).

### *In Vivo* Efficacy Studies

*In vivo* efficacy studies were performed by Pharma Models LLC using female immunodeficient nude mice (5–6 weeks old, 22–24 g) obtained from The Jackson Laboratory. All animal handling and experimental procedures were reviewed and approved by Institutional Animal Care and Use Committee (IACUC) prior to execution. The animals were maintained in accordance with institutional IACUC guidelines and housed under specific pathogen-free conditions with ad libitum access to food (Envigo 2920X) and sterile water.

For xenograft establishment, 1 × 10^6^ CAL33 or DETROIT562 cells suspended 1:1 in serum-free medium and Matrigel (100 μL total volume) were injected directly into the left flank of the mice. When tumors reached approximately 100 mm^3^, mice were randomized into different groups (n = 8-10/group) and treatment was initiated on the same day as randomization. The animals were administered the vehicle control or test article via intraperitoneal injection based on individual body weight for 28 days, with study endpoints of the loss of over 20% of body weight or a tumor volume exceeding 1500 mm^3^. Tumor dimensions were measured three times weekly using calipers, and volumes were calculated using the formula $\left(L\times W^{2}\right)∕2$. Body weight and clinical conditions were monitored daily. Animals were euthanized at the end of the study in accordance with institutional IACUC guidelines. Tumors were harvested, cleaned, weighed and photographed for analysis. Tumor growth differences were analyzed by one-way analysis of variance (ANOVA), and data are presented as mean ± SEM.

### *In Vivo* Tolerance Studies

*In vivo* tolerance studies were conducted at Frederick National Laboratory for Cancer Research (Frederick, MD) using female CD-1 mice (6–8 weeks old, 22–24 g) obtained from Charles River Laboratories. All animal handling and experimental procedures were performed according to Animal Care and Use committee guidelines (ACUC). Frederick National Laboratory for Cancer Research is accredited by American Association for Accreditation of Laboratory Animal Care (AAALAC) International and follows the Public Health Service Policy for the Care and use of Laboratory Animals. Animal care was provided in accordance with the procedures outlined in the “Guide for Care and Use of Laboratory Animals” (National Research Council; 2011; National Academies Press; Washington, D.C.).

CD-1 mice were acclimated for a minimum of 3 days before study initiation. Mice were administered the vehicle control or test article via intraperitoneal injection based on individual body weight (n=3). For the assessment of blood glucose levels, 1–2 drops of blood were taken from the tails of the mice and measured using an Accu-Chek Guide Me glucometer (Roche) at the indicated time point before or after treatment. Body weight and clinical conditions were monitored daily, with study endpoints of the loss of over 20% of body weight.

### Pharmacokinetic Studies

Female CD-1 mice were divided as three mice per group (per time point). Mice were dosed with cetuximab or PAC-XL as single intraperitoneal injections (100 mg/kg). Whole-blood sample was collected from cardiac puncture at selected times at set time points. After blood collection, blood was then centrifuged at 10,000 g for 5 min at 4 °C and the plasma fraction was separated and collected in 1.5 mL Eppendorf tubes and stored at −80 °C until analysis.

The concentration of total antibody was analyzed by ELISA method. The 96-well plates were coated with 1.0 μg/mL human EGFR protein at 4 °C overnight. After blocking with Super Block^™^ blocking buffer (ThermoFisher Scientific), plasma samples were added after 10,000-fold dilution with ELISA buffer (calibration standard: 70 – 1.0 ng/mL). The samples were incubated for 1 h at 37 °C, and then, the wells were washed three times with TBS-T and were incubated with an anti-human IgG (Fab-specific)-alkaline phosphatase-goat (Sigma-Aldrich) at 37 °C for 2 h. The KPL BluePhos^®^ Microwell Phosphatase Substrate (SeraCare) was added to the wells for color development. The optical density (OD) was read at 620 nm using a microplate reader.

The concentration of free BGT226 in plasma was determined by HPLC–QTOF–MS. Samples (50 μL) were diluted with 50 μL of acetonitrile and centrifuged at 15,000 rpm for 10 min. The supernatants were analyzed by HPLC–QTOF–MS and characterized with the small molecular method described previously. The percentage of released BGT226 was quantified based on a calibration curve of BGT226 prepared in PBS (10 mM, pH 7.2) and acetonitrile (1:1, v/v) as shown above.

### Statistical analysis

The statistical analysis and graphic presentations were performed with Graphpad Prism 10 software. Data points are displayed as the mean ± SD or mean ± SEM. The statistical significance of the differences between the two groups was determined using the 2-tailed Student’s t-test, and multiple groups were compared using a one-way analysis of variance (ANOVA) test. P values of < 0.05 were accepted as a significant difference. (*P < 0.05; **P < 0.01, ***P < 0.001, ****P < 0.0001).

## Extended Data

## Supplementary Material

This is a list of supplementary files associated with this preprint. Click to download.

• PACXLSI.docx

## Data Availability

Further information on research design is available in the Nature Research Reporting Summary linked to this article. Source data are provided with this paper. All other data supporting the findings of this study are available from the corresponding author on reasonable request.
